# The Submerged Dyslexia Iceberg: How Many School Children Are Not Diagnosed? Results from an Italian Study

**DOI:** 10.1371/journal.pone.0048082

**Published:** 2012-10-31

**Authors:** Chiara Barbiero, Isabella Lonciari, Marcella Montico, Lorenzo Monasta, Roberta Penge, Claudio Vio, Patrizio Emanuele Tressoldi, Valentina Ferluga, Anna Bigoni, Alessia Tullio, Marco Carrozzi, Luca Ronfani, CENDi (National Committee on the Epidemiology of Dyslexia) working group, and for the Epidemiology of Dyslexia of Friuli Venezia Giulia working group (FVGwg)

**Affiliations:** 1 Epidemiology and Biostatistics Unit, Institute for Maternal and Child Health - IRCCS “Burlo Garofolo”, Trieste, Italy; 2 Child Neurology and Psychiatry Ward, Institute for Maternal and Child Health - IRCCS “Burlo Garofolo”, Trieste, Italy; 3 Child Neuropsychiatry Department, UOC NPI B La Sapienza University, Rome, Italy; 4 Child Neuropsychiatry Unit, Hospital of San Donà di Piave, San Donà di Piave, Italy; 5 Psychology Department, University of Padua, Padua, Italy; University of Leicester, United Kingdom

## Abstract

**Background:**

Although dyslexia is one of the most common neurobehavioral disorders affecting children, prevalence is uncertain and available data are scanty and dated. The objective of this study is to evaluate the prevalence of dyslexia in an unselected school population using clearly defined and rigorous diagnostic criteria and methods.

**Methods:**

Cross sectional study. We selected a random cluster sample of 94 fourth grade elementary school classes of Friuli Venezia Giulia, a Region of North Eastern Italy. We carried out three consecutive levels of screening: the first two at school and the last at the Neuropsychiatry Unit of a third level Mother and Child Hospital. The main outcome measure was the prevalence of dyslexia, defined as the number of children positive to the third level of screening divided by the total number of children enrolled.

**Results:**

We recruited 1774 children aged 8–10 years, of which 1528 received parents’ consent to participate. After applying exclusion criteria, 1357 pupils constituted the final working sample. The prevalence of dyslexia in the enrolled population ranged from 3.1% (95% CI 2.2–4.1%) to 3.2% (95% CI 2.4–4.3%) depending on different criteria adopted. In two out of three children with dyslexia the disorder had not been previously diagnosed.

**Conclusions:**

This study shows that dyslexia is largely underestimated in Italy and underlines the need for reliable information on prevalence, in order to better allocate resources both to Health Services and Schools.

## Introduction

Developmental dyslexia, or specific reading disability, is defined as an unexpected, specific, and persistent failure to acquire efficient reading skills despite conventional education, adequate intelligence, and socio-cultural opportunity. [1], [2] Furthermore, this disability significantly interferes with scholastic learning or with daily activities that require reading ability.

Although dyslexia is one of the most common neurobehavioral disorders affecting children, prevalence is uncertain and available data are scanty and dated. Studies conducted in English-speaking countries, mainly in the Nineties, show prevalences ranging from 5 to 17.5%.[3]–[5] This range is due to the different diagnostic methods and definitions used [4], [6], [7]. For example, in a population based retrospective birth cohort study, the cumulative incidence rates of dyslexia by the age of 19 years varied from 5.5 to 11.8% depending on the formula used for diagnosis. [8] The geographical setting could also have a role. Dyslexia was found to occur in 9.9% of ten-year-old children living in a metropolitan area vs. 3.9% of children living in small towns. [9].

In Italy, a limited number of studies shows lower prevalences but confirms uncertainty (from 1.3% to 8.5%).[10]–[18] In the Sixties, in children aged 8 to 10 years living in a metropolitan area of Northern Italy, a prevalence of dyslexia of 1.34% was found using a collective reading test and a spelling test. [11] In studies conducted subsequently adopting the same instruments, on larger and more heterogeneous population samples, higher prevalence was reported, ranging from 3% to 6.5%.[12]–[14] A prevalence of dyslexia of 3.4% has been reported in a sample of children aged 8 to 10 years, that were first screened by teachers and subsequently underwent reading and writing tests [15] and of 5% in a sample of children of the same age that underwent collective tests on word/non word recognition, word/non word dictation, MT battery. [16] The prevalence of dyslexia estimated in a sample of high school children in Central Italy was 6.5%. [17] A longitudinal study conducted on children of elementary and middle schools living in Isola d’Elba (Tuscany) reported a prevalence of learning disabilities ranging from 0.88 to 1.23% in different school years from 1991 to 1999. [18] A cross national comparison of dyslexia prevalence in Italy and in the United States showed that dyslexia has a higher prevalence in the United States than in Italy and confirmed that the use of different methods and diagnostic criteria actually led to different values of dyslexia prevalence (3.6 to 8.5% in Italy vs. 4.5 to 12.0% in the United States). [19].

As described above, the variability in dyslexia prevalence estimates could be due to the different methods and tests adopted for diagnosis, the type of disability evaluated (i.e. dyslexia vs. learning disabilities), the different age ranges considered, the different geographic setting evaluated, as well as the different language spoken by the children. The key element to be taken into account is the definition of dyslexia adopted. [6] In Italy, an attempt to clearly define diagnostic criteria for dyslexia only dates back to 2007. These criteria were defined during the Montecatini Consensus Conference [20] by a panel of experts from Italian associations and institutions involved with children with learning disabilities, and were revised in 2011 (Text S1). [21], [22] In 2010 the Italian Government approved a law to guarantee access to equal educational opportunities for students with learning disabilities (Italian Law n° 170, 8 October 2010). [23] Thus, Italian studies conducted before 2007 suffer from the lack of univocal diagnostic criteria that obviously influence reported prevalence data.

The lack of recent and reliable data on dyslexia prevalence leads to negative effects at the cultural (unmeasured phenomena can be ignored, underestimated or overestimated), clinical (insufficient resources for diagnosis and rehabilitation), and pedagogical level (insufficient resources for schools). Moved by these considerations, associations and institutions (listed in Annex) established a National Committee (CENDi) with the aim to define and apply methods and instruments and to conduct regional researches on the prevalence of dyslexia in representative unselected school populations. This paper describes in detail the methodology adopted and the results of the field study conducted in the Region of Friuli Venezia Giulia of Italy.

## Materials and Methods

Cross sectional study carried out in Friuli Venezia Giulia (FVG), a region of North Eastern Italy. The study was approved by the Independent Bioethics Committee of the Institute of Maternal and Child Health - IRCCS “Burlo Garofolo”, Trieste, Italy.

### Sample

Children aged 8–10 years attending the 4^th^ year of Italian primary schools were enrolled. In Italy, primary school includes children from age six to eleven and is organized in five school years. The 8–10 years of age range was chosen, in accordance with the Montecatini Consensus Conference, [20] for the following reasons: 1) by this age most learning delays are spontaneously resolved; [24], [25] 2) a narrow age range reduces the number of developmental variables to be controlled; 3) in this age range, reading compensation strategies are not fixed, so the detection of reading difficulties should be easier.

Exclusion criteria were: 1) children with certification of mental retardation formulated by local health authorities according to the Italian Law n° 104/92 (framework law on disabled persons). [26] The exclusion of children with mental retardation was recommended by the Montecatini Consensus Conference, [20] and is in agreement with the definitions given by ICD-10 and DSM-IV. [1], [2] 2) non-Italian nationality; 3) absence from school for more than two months since the first grade.

Bilingual children of Italian nationality and those with chronic diseases not related with learning abilities were not excluded. Pupils with previous diagnosis of dyslexia were included and re-evaluated.

To determine the sample size, the total population of children attending grade four in the Region (9687 children) was considered. A sample size of 1500 children was estimated, hypothesizing a prevalence of dyslexia of 4%, ranging from 3% to 5%, with a precision of 5% and a power of 80%. An extra 15% were enrolled to compensate possible drop outs. Based on the average number of children per class, we decided to randomly extract clusters of grade four classes rather than children. 94 classes were thus randomly extracted, comprising 1774 children.

### Identification of Children with Dyslexia and Writing Disorders

The field study started in September 2008, at the beginning of the school year, and ended in October 2009, with the third level evaluation. Parents were adequately informed of the aim of the research and signed the informed consent. To identify children with reading disability, three consecutive levels of evaluation were carried out: the first two directly at school by specifically trained psychologists, the last in the Child Neuropsychiatry Unit of a third level hospital (IRCCS “Burlo Garofolo” of Trieste).

#### First level evaluation

Considering the large number of children to be screened, the first level evaluation was carried out through collective tests and questionnaires. To avoid discriminations, all children in the selected classrooms were tested, and exclusion criteria were applied afterwards. The evaluation was based on the following tools:

A short anamnestic questionnaire filled in by parents, with questions concerning the child and his/her family (language spoken at home, age, work, parents’ qualifications, health status of the child, handicap certification and previous diagnosis of Learning Disability).
A specific questionnaire, filled in by the classroom teachers for each child of the selected classes, in order to detect Learning Disabilities (LD). This tool was derived from the validated questionnaire “RSR-DSA. Questionario per la rilevazione di difficoltà e disturbi dell’apprendimento”. [27] Since this questionnaire includes 52 items investigating all LDs, for the purpose of this study, 34 specific questions pinpointing dyslexia and closely related disorders (difficulties in math, handwriting, spelling, and reading) were extracted, in agreement with the questionnaire’s authors. Answers were scored on a 0 to 3 point scale (0 = never; 1 = sometimes; 2 = often; 3 = always). Children were considered having reading difficulties if: 1) the total score ≥85° percentile or 2) the score on two subgroups of questions specifically addressing dyslexia ≥90° percentile. The diagnostic accuracy of these criteria was tested on a sample of 200 children previously enrolled (100 with diagnosis of dyslexia and 100 controls without reading difficulties) (data not published). The results showed a sensitivity of 82% and a specificity of 100%, with 91% of children correctly classified. The diagnostic accuracy was considered good and comparable with that obtained by more complex screening instruments. [28]
A dictation task for the forth grade, derived from the “BVSCO – Battery for the assessment of writing skills in children from 7 to 13 years old”. [29] To avoid bias due to different reading styles and timing during dictation, the task was recorded on CD and played in the classrooms using a laptop with adequate amplification. The use of a dictation test to screen reading difficulties is supported by studies on the comorbidity between reading and spelling difficulties. [30] Children scoring ≥90° percentile were considered as having learning difficulties, as indicated by the normative data of the tests used. [29]

**Figure 1:**
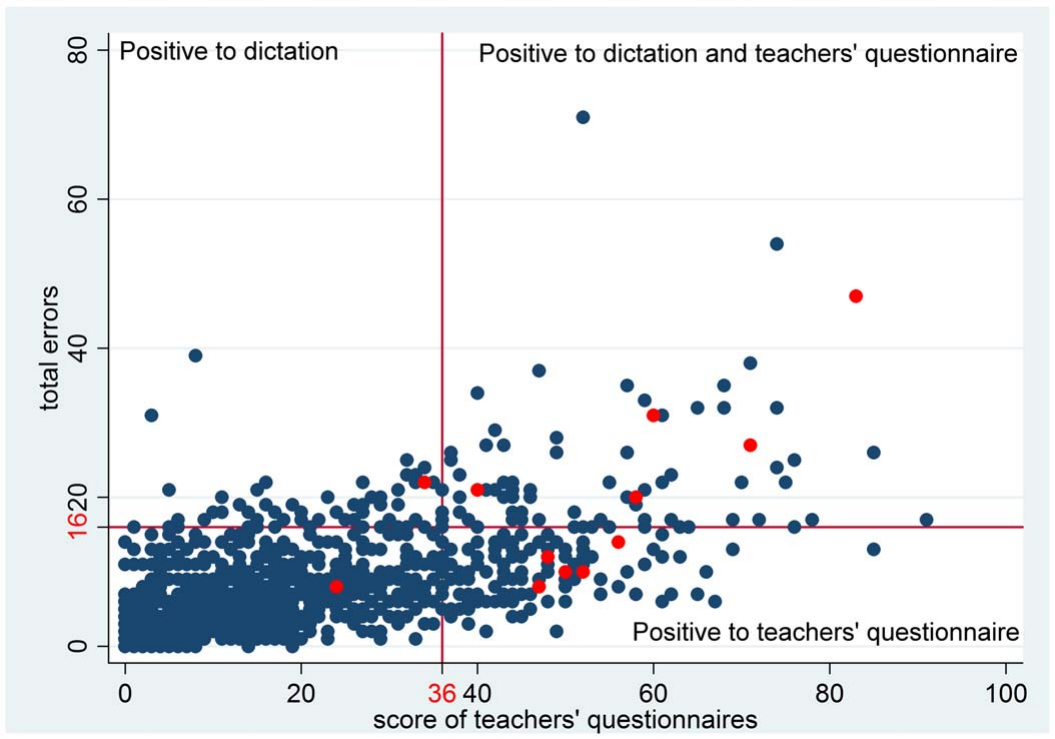
Children positive to the criteria of the first evaluation level. In red children with previously formalised diagnosis of dyslexia.

After applying inclusion/exclusion criteria, children who scored positive by the teachers’ questionnaire and/or in the dictation task were selected for the second level evaluation. These criteria allowed to correctly identify all 13 children who had already received a formal diagnosis of dyslexia, except one (Figure 1). This child, however, had just completed a rehabilitation programme before the beginning of the school year which explains his good performance in the tests.

To ensure that all children with reading difficulties were identified, teachers were further asked to indicate 1) children who read more slowly than classmates 2) children who made more reading errors than classmates. All children identified by at least one of these two additional criteria were also selected for second level testing. Furthermore, when possible, in classes in which children with reading difficulties were identified, classmates with adequate performance were also randomly selected for second level evaluation, in order to avoid discrimination from peers.

#### Second level evaluation

The aim of the second level was to identify children with reading difficulties and adequate cognitive ability through individual tests. Evaluations were conducted at school and carried out by the same researchers involved in the first level evaluation (specifically trained psychologists). The following tools were used for individual testing:

word and non word reading tasks derived from the DDE-2 Battery (Battery for the assessment of Developmental Dyslexia and Dysorthographia-2) [31] were used to assess reading speed and accuracy. These tasks provided a total of 4 scores (2 for accuracy and 2 for speed). Children were considered to perform poorly if: a) they failed in at least one of four scores (cut-offs: z-score ≤−1.8 standard deviations from the mean for speed scores, a score <5th percentile in the accuracy scores), or b) they failed in at least two of the four scores (cut-offs: z-score ≤−1.5 standard deviations from the mean in speed scores, a score <10th percentile in accuracy scores). The cut-offs selected for the criterion *a* were those indicated by the normative data of the DDE-2 Battery; [31] for the criterion *b*, the CENDi chose less selective cut-offs in order to reduce false negatives to the minimum and to also identify children with mild disorders.
Vocabulary and Block Design subtests of the WISC-III (Wechsler Intelligence Scale for Children) [32], [33] to estimate child’s cognitive ability. Since the time at school was insufficient to perform the entire WISC-III scale, these two subtests were selected as the more related to the child’s IQ score. [32], [33] A weighted score ≤7 in both sub-tests identified a poor IQ, in agreement with those reported in the WISC-III manual.

The second level criteria were defined by CENDi according to the Montecatini Consensus Conference. [20] The selected tests and cut-offs allowed to correctly identify all children with a prior formal diagnosis of dyslexia, except one. Again, this child had just completed a rehabilitation programme and showed a marked improvement in performance.

Children with adequate WISC-III subtest scores, yet poor performance in reading tasks, were selected for the third level of screening.

### Third Level Evaluation

The aim of the third level was to confirm the diagnosis of dyslexia. To avoid bias in the diagnostic process, all children selected at the second level evaluation were referred and evaluated at the Child Neuropsychiatry Unit of the third level hospital IRCCS “Burlo Garofolo”. All children were assessed as follows:

- Detailed questionnaire (see Text S2) filled in by parents and checked during an interview with a psychologist. This standardized protocol allowed to collected anamnestic information about the child’s development (speech, gait, autonomy, etc.), kindergarten and primary school attendance (social skills and communication, presence of learning disabilities, etc.), clinically significant events that may have occurred during childhood (i.e. injuries, illnesses) and information regarding formal education of close relatives (school performance, learning difficulties, etc.)
- Raven’s Progressive Matrices PM47 to evaluate in a short time the cognitive performance. [34]
- MT battery (Prove di lettura MT per la scuola elementare-2) to evaluate speed and accuracy of text reading. [35], [36]
- DDE-2 (Battery for the evaluation of Dyslexia and dysorthography-2) to evaluate speed and accuracy of word and non word reading (tasks 2 and 3) and accuracy of spelling (tasks 6 and 7). [31]
- Strengths and Difficulties Questionnaire (SDQ) administered to parents to evaluate the mental health status of their child. [37]

To confirm the diagnosis of dyslexia we took into account:

Six scores (three on accuracy and three on speed) derived from the reading tests (test MT and tasks 2 and 3 from battery DDE-2).
Parents responses to two questions derived from the detailed questionnaire completed during the third level evaluation: A) presence of early specific reading and/or spelling difficulties B) the child is not independent in performing homework.
Teachers’ positive responses to eight specific questions on decoding skills derived from the questionnaire compiled during the first level evaluation. [27] These items relate to accuracy (skips lines reading, makes more errors in reading than her/his classmates, replaces, omits, adds or reverses letters, makes up words, is fast but incorrect in reading) and speed (the child reads more slowly than her/his peers).

Combining the results of these tests, CENDi experts defined three possible criteria to guide the diagnosis of dyslexia (each criterion requires that all the defined conditions be met):

Poor performance (Table 1) on reading tasks in at least three out of six scores.
a) Poor performance (Table 1) on reading tasks in at least two scores out of six, in two different reading tests (the poor performance should not refer to two parameters in a single reading test); AND b) parental acknowledgement of the disorder by a positive response to at least one of the two questions A and B of the detailed questionnaire.
a) Poor performance (Table 1) on reading tests in at least three out of six scores; AND b) parental recognition of the disorder by a positive answer to both questions A and B of the detailed questionnaire; AND c) teachers’ acknowledgement of the disorder by a positive response to at least half of the questions selected by the teachers’ questionnaire.

**Table 1:** Definition of poor performance in reading tasks by diagnostic criteria defined at the third level evaluation.

**First and second criteria**	
**DDE-2** *	z-score ≤−1.8 (speed)
	OR
	percentile <5° (accuracy)
**MT** §	z-score ≤−2 (speed)
	OR
	percentile ≤5° (accuracy)
**Third criterion**	
**DDE-2** *	z-score between −1.8 and −1.5 (speed)
	OR
	percentile between 5° and 10° (accuracy)
**MT** §	z-score between −2 and −1.5 (speed)
	OR
	between 5° and 10°
**Fourth criterion**	
**DDE-2 non word** *	z-score ≤−1.8 (speed)
	OR
	percentile ≤5° (accuracy)

* DDE-2: Battery for the evaluation of Developmental Dyslexia and Dysorthography-2.

§ MT: MT battery (Prove di lettura MT per la scuola elementare-2).

The cut-offs reported in Table 1 were defined by the CENDi experts, according to the normative data and to the recommendations of the Montecatini Consensus Conference. [20] For the third criterion the CENDi decided to choose less selective cut-offs on a higher number of scores, together with the recognition of difficulties by parents and teachers, in order to identify children with a less pronounced form of disability.

An additional fourth criterion was defined to identify children with only phonological difficulties: a) poor performance (Table 1) in at least one score on non word reading tasks; AND b) parental recognition of the disorder by a positive answer to both questions A and B of the detailed questionnaire; AND c) teachers’ recognition of the disorder by a positive response to at least half of the questions in the teacher questionnaire. The rationale of this criterion is based on the evidence that children with dyslexia may have difficulties in phonological recoding when measured with non word reading tests. [38].

Children identified by the fourth criterion were considered separately in the prevalence evaluation since CENDi experts considered this criterion weaker than the previous. Any case of discrepancy between the results obtained applying the criteria and the clinician’s judgement was also considered separately (i.e. children who were diagnosed dyslexic according to the criteria but not according to the clinician’s evaluation and, conversely, children who were diagnosed according to clinician assessment but not according to the criteria). The clinical history of these children and the third level evaluation tests were reviewed by the CENDi experts to confirm or deny the diagnosis.

Children identified by the criteria as dyslexic were subjected to further evaluations: a) Completion of the scale WISC-III; [32], [33] b) Math tasks (numerical facts, count backwards, numbers dictation) from the ABCA test; [39] c) the Italian adaptation of the Multidimensional Self-Concept Scale (MSCS); [40] d) Questionnaire “A chi assomiglio”, [41] the Italian adaptation of the “Self-Perception Profile for Children”; [42] e) neurological objective examination. The results of the tests applied at points *b*, *c*, *d* were not used for the purpose of this paper and consequently are not presented in this manuscript.

Based on the results of these additional evaluations and on data reported in detailed parents questionnaire, children were considered as non-dyslexic if they had: a) a cognitive delay defined by the results of the WISC-III scale (scores on Verbal IQ and Performance IQ <85); b) diseases or sensory and neurological abnormalities; c) severe psychopathology; d) conditions of environmental, social or cultural disadvantage; e) poor teaching.

Children with suspected neuropsychiatric problems were referred to the Neuropsychiatry Unit for further investigations.

**Figure 2:**
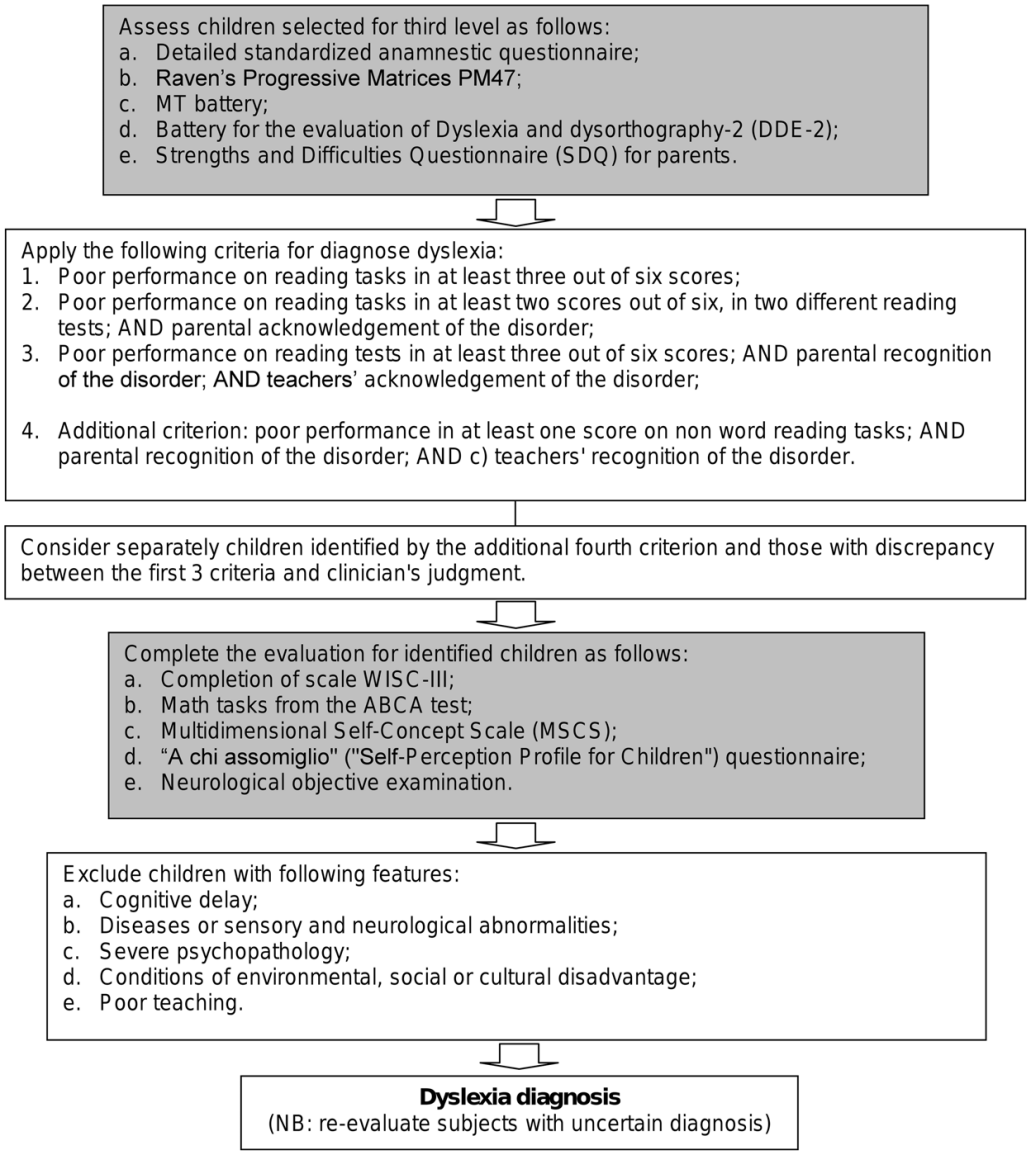
Steps for third level evaluation.

Figure 2 summarizes the procedure for the third level of screening.

### Statistical Analysis

Continuous data were presented as means and standard deviations, categorical data as absolute frequencies and percentages.

Prevalence of dyslexia was defined as the number of children positive to the third level of screening (numerator) divided by the total number of children analysed at the first level.

Two values of prevalence were calculated, the first including only cases with diagnosis of dyslexia according to the first three criteria defined by CENDi, and the second also including cases with uncertain diagnosis (defined by the 4^th^ criterion or by discrepancy in diagnosis between criteria and clinical judgment).

To predict the diagnosis of dyslexia in children lost to the third level follow up, a multinomial logistic regression analysis was carried out. The estimation process included the results of the tests conducted at the second level (speed and accuracy in word and non word reading tests) as predictors and the diagnosis of dyslexia as the dependent variable. A step-down procedure was applied to the saturated model retaining only variables with a p-value <0.05. The final logistic model was used to predict the probability of being dyslexic, and ROC (Receiver-Operating-Characteristic) analysis was used to identify the cut-off correctly classifying the highest rate of subjects. Based on this cut-off, subjects lost to the third level follow up were classified as with or without dyslexia and included in the calculation of the total prevalence.

## Results

A total of 94 grade four classes were selected using random cluster sampling. Overall, 1774 pupils were contacted, and for 1528 of them parents gave their consent to participate.

### First Level Evaluation

**Figure 3:**
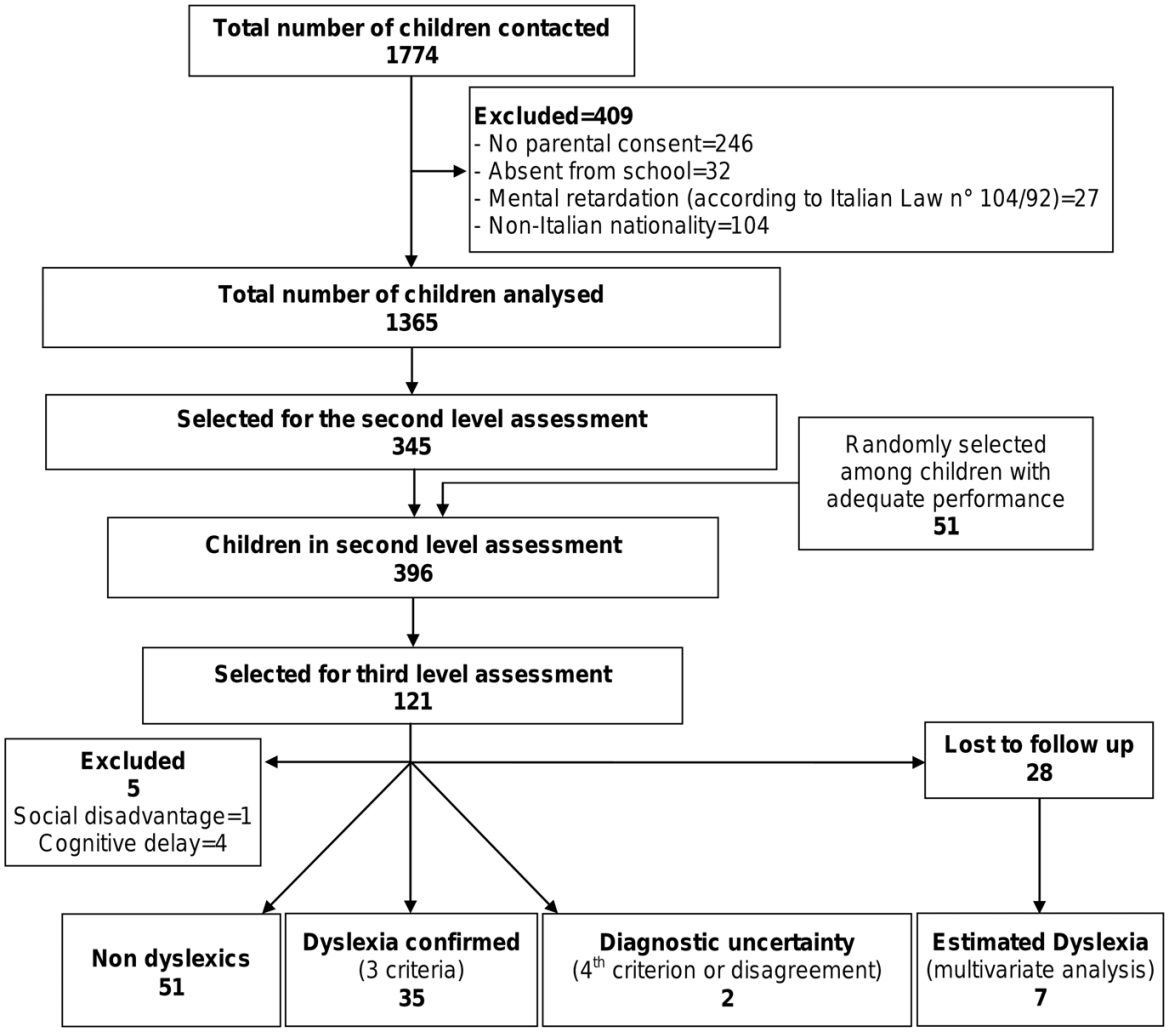
Study flow chart.

Thirty-two children were absent from school on days when the assessments were carried out. The remaining 1496 children participated in the first level evaluation. After application of exclusion criteria, 1365 children were included in the sample and their performance was analysed (Figure 3 and Figure S1). Characteristics of these children are presented in Table 2. Thirteen out of the 1365 children (1%) had already received a formal diagnosis of dyslexia.

**Table 2:** Main characteristics of the selected population.

Variable		Children (n = 1365)
Sex	Female	663 (48.6%)
	Male	702 (51.4%)
Language spoken at home	Italian	1256 (92.0%)
	Dialect	105 (7.7%)
	Other	4 (0.3%)
Age of the mother, mean (standard deviation)		40.1 (4.71)
Age of the father, mean (standard deviation)		43.2 (5.44)
Mother’s formal education level	None/elementary	20 (1.4%)
	Lower secondary	413 (30.4%)
	Upper secondary	729 (53.6%)
	Degree	198 (14.6%)
Father’s formal education level	Elementary	22 (1.6%)
	Lower secondary	511 (38.0%)
	Upper secondary	634 (47.2%)
	Degree	177 (13.2%)
Mother with job		1053 (77.4%)
Father with job		1319 (98.2%)
Children with previously formalised dyslexia diagnosis		13 (1.0%)
Children with previously formalised Learning Disabilities diagnosis (dyslexia, dysgraphia, dysorthography, dyscalculia)		27 (2.0%)

Two hundred eighty-three children scored positive in at least one of the two tests of the first level (dictation or teachers’ questionnaire) and 62 were identified by the additional questions addressed to the teachers.

### Second Level Evaluation

Overall, 396 children were selected for the second level assessment (345 selected at the first level of screening and 51 randomly selected from children with adequate performance). One hundred twenty-one scored positive in the tests and were selected for the third level of screening.

### Third Level Evaluation

Ninety-three of 121 children underwent further testing to confirm diagnosis. Parents of 28 children refused to continue in the study. Five children of 93 were excluded since they met the third level exclusion criteria (4 with cognitive delay and 1 with social disadvantage). Of the remaining 88 children, 51 were classified without dyslexia, 35 were diagnosed with dyslexia, 1 was positive only according to the 4^th^ criterion and 1 was positive on the basis of the defined criteria but the clinician did not confirm the diagnosis.

The logistic model built for predicting dyslexia in children lost to the third level follow up retained as significant predictors the speed z-score of the word reading test (adjusted OR 0.13, 95% CI 0.02–0.87 and adjusted OR 0.07, 95% CI 0.01–0.61 respectively) and the speed z-score of the non word reading test (adjusted OR 1.29, 95% CI 1.11–1.51).

The ROC (Receiver-Operating-Characteristic) curve was generated with the predicted probability of the logistic model. The resulting area under the curve was 0.93. The cut-off with the highest percentage of correctly classified (86.2%), gave us a sensitivity of 70.3% and a specificity of 98.0%. Using this cut-off, 7 out of 28 children were considered to be dyslexics.

The prevalence of dyslexia including children who fell in the three diagnostic criteria (n = 35) and children estimated with the logistic model (n = 7) was 3.1% (42/1365) (95% CI 2.2–4.1); adding the child positive to the fourth criterion and that with uncertain diagnosis the prevalence rose to 3.2% (95% CI 2.4–4.3).

## Discussion

This study accurately estimated the prevalence of dyslexia in a representative and unselected grade four school population. Prior to the study, out of 1365 children screened, 13 (1%) had a formal diagnosis of dyslexia and 27 (2%) of Learning Disabilities (including dyslexia, dysgraphia, dyscalculia and dysorthography). These findings are similar to those available for other areas in Italy. [43]

The prevalence of dyslexia at the end of the study rose to 3.1–3.2%. Therefore, according to our study, dyslexia is not recognized in two out of three children aged 8–10 years, when the disorder should be clearly expressed and identified. This discrepancy calls for justifications, especially in a Region like FVG which is generally considered quite sensitive to this issue and where attention to the disorder has been recently shown both by Schools and Health Services through workshops, conferences, meeting, circulation of guidelines, and interventions in schools. Since dyslexia has been identified as a specific risk factor for increased internalizing, anxiety and depressive behaviours, [44], [45] suicidal ideation, and school failure and drop out, [46] especially during adolescence, early diagnosis and intervention appear to be crucial for the affected children.

Given the differences in methods, definitions and diagnostic criteria adopted, it is difficult to compare the prevalence data obtained in this study with those previously reported in Italy.[10]–[18] However, our results are in line with the expected for Italy. The comparison with studies conducted in English-speaking countries is even more complex, as the frequency of dyslexia differs between languages. As addressed in a recent literature review, the neural basis of dyslexia is similar across different languages. However, cross-cultural specificities were evident in the manifestations of dyslexia, as clinically significant difficulties are less common in transparent languages with consistent orthographies (i.e. Italian), if compared to languages with inconsistent orthographies (i.e. English). [6] Poor readers in English, French and Italian showed similar patterns of aberrant neural activation, but Italian subjects present a higher reading accuracy. [47] A cross national comparison of dyslexia prevalence in Italy and in the United States confirmed that dyslexia is more prevalent in the United States than in Italy. Furthermore, in this cross national study, different diagnostic criteria for dyslexia were applied to the enrolled children, thus achieving different values of dyslexia prevalence (3.6 to 8.5% in Italy vs. 4.5 to 12.0% in the United States). [19]

Consequently, our estimate of prevalence cannot be extended to different contexts, in particular to populations speaking less phonetically regular languages, like English. However, research on dyslexia has historically focused mainly on the English language, and reliable data on other languages are required to better understand cross-cultural differences and similarities. [6], [48] Furthermore, given the important consequences it might have on dyslexic children and on allocation of resources, it would be interesting to understand whether the problem of underestimation of dyslexia prevalence is common to population speaking other languages. The available scientific literature does not allow us to answer this question and ad hoc studies, designed and conducted following clearly defined rigorous methods would be required, in order to guarantee their reproducibility and a clear comprehension of what is being assessed. In our study, to confirm the diagnosis of dyslexia, a detailed and unequivocal diagnostic algorithm, combining the different tests and the cut-offs indicated by the Montecatini Consensus Conference (see Text S1) [20] was developed by the CENDi, thus contributing to a more precise definition of the diagnostic criteria of dyslexia in Italy. The study presents several other strengths: the use of a cluster sampling procedure to enrol a representative sample of regional school pupils aged 8–10 years; the large sample size (about 15% of children attending grade four in Friuli Venezia Giulia); the rigorous application of screening tools by a specifically trained staff; the third level assessment (confirmation of diagnosis) performed in a single third level Neuropsychiatry Unit to avoid bias in the diagnostic process; the involvement in this process of both child neuropsychiatrists and psychologists.

The study has some limitations: given the large sample size, it was not possible to administer individual reading tests to all children at the first level evaluation. Consequently, the CENDi decided to adopt two different tools: a shortened version of a validated questionnaire to be filled in by teachers for each child in the selected classrooms; [27] a dictation test collectively administered to children in a standardized manner. [29] The use of a dictation test to screen reading difficulties is supported by studies on comorbidity between reading and spelling difficulties. [30] Since these tools have never been used before in screening studies for dyslexia, their use could have resulted in a loss of children with reading difficulties at the first level screening (false negatives). Nevertheless, we believe we have kept this risk to the minimum, considering that: a) children were selected for second level if scores were positive even in one of the two tools; b) a low cut-off (85^th^ percentile) for the teachers’ questionnaire was selected to include a large number of children; c) the criteria adopted allowed to correctly identify all 13 children who had already received a formal diagnosis of dyslexia, except one. However, the non-selected child completed a rehabilitation treatment just before the beginning of the study and this should explain the improvement in test performance; d) in addition to the questionnaire, teachers were asked to further point out children with reading difficulties through two simple and standardized questions; children identified by at least one of these two additional criteria were selected for second level analysis, despite results of teachers’ questionnaire and dictation tests.

### Conclusions

This study shows that the tools and the methodology defined by the CENDi are applicable and allow to estimate the prevalence of dyslexia in an unselected grade four school population in Italy. In our setting the prevalence ranged from 3.1 to 3.2%, in line with expectations. Therefore, it is interesting to notice that in two out of three children with dyslexia the disorder had not been previously diagnosed. These data confirm the need for reliable information on dyslexia prevalence in order to better allocate resources both to Health Services and Schools. Finally, this study provides original and up-to-date information, useful in the debate on trans-cultural features of dyslexia.

## Supporting Information

Figure S1 **Details on children included and excluded at first and second level evaluation.**
(PDF)

Text S1 **General criteria for dyslexia diagnosis defined by the Montecatini Consensus Conference (2007) and by the subsequent national Consensus Conferences (2011).**
(PDF)

Text S2 **Standardized questionnaire filled in by parents.**
(PDF)
