# Supplementary figures and images for: The Submerged Dyslexia Iceberg: How Many School Children Are Not Diagnosed? Results from an Italian Study
Source: PLoS One. 2012 Oct 31;7(10):e48082. doi: 10.1371/journal.pone.0048082 (PMC3485303; doi:10.1371/journal.pone.0048082)

**Figure S1. Details on children included and excluded at first and second level evaluation**

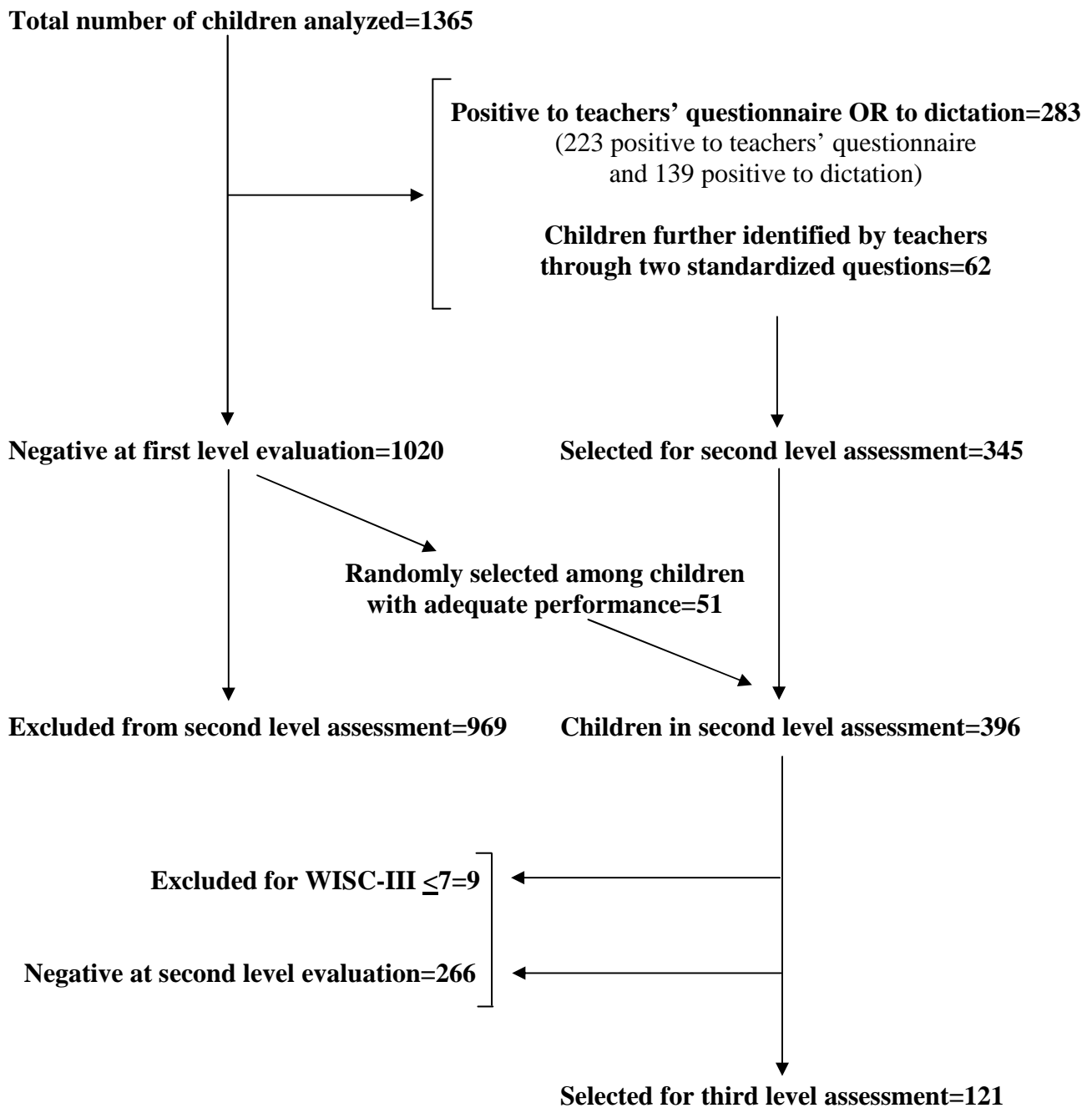

Supplement: Figure S1 — Details on children included and excluded at first and second level evaluation. (PDF) [file pone.0048082.s001.pdf]
