# Supplementary material for: The Submerged Dyslexia Iceberg: How Many School Children Are Not Diagnosed? Results from an Italian Study
Source: PLoS One. 2012 Oct 31;7(10):e48082. doi: 10.1371/journal.pone.0048082 (PMC3485303; doi:10.1371/journal.pone.0048082)
Supplement: Text S1 — General criteria for dyslexia diagnosis defined by the Montecatini Consensus Conference (2007) and by the subsequent national Consensus Conferences (2011). (PDF) [file pone.0048082.s002.pdf]

Text S1. General criteria for dyslexia diagnosis defined by the Montecatini Consensus Conference (2007) [1] and by the subsequent national Consensus Conferences (2011) [2,3]

1) Exclusion of conditions that might influence the results of the test, such as:
   a. major sensory and neural disabilities;
   b. significant emotional disorders;
   c. environmental situation of socio-cultural disadvantage that can interfere with adequate formal education;

2) Intelligence quotient within normative limits: in case of adoption of single-dimensional tests (for example: Raven's Progressive Matrices), IQ not less than -1 standard deviation (equivalent to a score of 85) with respect to mean expected values by age; in case of multi-dimensional tests (for example: WISC-III scale), at least one single-dimensional IQ not less than -1 standard deviation (equivalent to a score of 85);

3) Administration of standardised reading tests: letters, words, non words, reading text;

4) Joint evaluation of both parameters of speed and accuracy;

5) Need to establish a significant distance from the expected mean values for the grade attended by the child in one of the two parameters, conventionally established at -2 SD from the mean for speed and below the 5th centile for accuracy;

6) Minimum age for diagnosis not before the end of the second year of primary school.

References

1. Consensus Conference (2007) Disturbi evolutivi specifici di apprendimento. Available: www.lineeguidadsa.it/download_documentiDSA/RACCOMANDAZIONI_PRATICA_CLINICA_DSA2007.zip. Accessed: 01 September 2012.

2. PARCC (2011) DSA Documento d'intesa. Available: www.lineeguidadsa.it/download_documentiDSA/Raccomandazioni_clinica_DSA11.zip. Accessed: 01 September 2012.

3. Istituto Superiore di Sanità (2011) Consensus Conference Disturbi Specifici dell'Apprendimento. Sistema nazionale per le linee guida Ministero della salute. Available: www.lineeguidadsa.it/download_documentiDSA/Raccomandazioni_CC_DSA.zip. Accessed: 01 September 2012.
