# Supplementary material for: The Submerged Dyslexia Iceberg: How Many School Children Are Not Diagnosed? Results from an Italian Study
Source: PLoS One. 2012 Oct 31;7(10):e48082. doi: 10.1371/journal.pone.0048082 (PMC3485303; doi:10.1371/journal.pone.0048082)
Supplement: Text S2 — Standardized questionnaire filled in by parents. (PDF) [file pone.0048082.s003.pdf]

Text S2. Standardized questionnaire filled in by parents

Gent.le Sig/a,
le chiediamo se gentilmente può rispondere ad alcune domande relative alla storia passata di suo figlio/a ed alla vostra famiglia. Queste risposte saranno molto utili per orientarci nella diagnosi e serviranno come traccia all'operatore per un successivo eventuale approfondimento sulla natura delle difficoltà riscontrate.
Grazie

1. Primi anni di vita

1.1 Dopo quante settimane di gestazione è nato/a suo figlio/a? _____
1.2 Qual' era il peso alla nascita? _____ Kg 1.3 Si ricorda il punteggio APGAR al 5° minuto? ___
1.4 Il parto è stato: spontaneo senza ossitocina □ spontaneo con ossitocina □ taglio cesareo□
1.5 Il bambino/a è stato allattato al seno? Sì □ No □ Se sì, per quanti mesi? ____
1.6 Ripensando al primo anno di vita di suo figlio/a, ha qualche elemento o problema da rilevare rispetto al sonno (es: ritmi sonno veglia regolari o risvegli frequenti)? Sì □ No □
Se sì, quale/i? ______________________________________
1.7 Quando ha cominciato a dire le prime parole? ____° mese
1.8 Il bambino ha cominciato ad usare con maggiore facilità parole diverse e a ripetere parole nuove appena sentite: prima dei due anni □ dopo i due anni □
1.9 Quando ha iniziato ad abbinare due parole per formare delle frasi (es: mamma pappa, tata nanna)?
A 24 mesi □ a 30 mesi □ a 36 mesi □ dopo i 36 mesi □
1.10 Vi sono state difficoltà nell'acquisizione di una delle due lingue (*solo per bambini bilingui*)?
Sì□ No □ Se si, quale/i? ____
1.11 A che età ha iniziato a camminare da solo/a? ____° mesi
1.12 A che età ha tolto il pannolino di giorno? ___° anno E di notte? ____° anno
1.13 Qual è la mano che suo figlio/a preferisce usare? Destra □ Sinistra □ Entrambe □
1.14 Si ricorda all'incirca a che età il bambino ha imparato a usare le forbici?_____anni
1.15 Si ricorda all'incirca a che età ha imparato a fare il nodo delle scarpe o di altri capi di abbigliamento?_____anni

2. Scuola dell'infanzia:

2.1 All'ingresso della scuola dell'infanzia, il bambino si faceva capire da tutti, anche da chi non lo conosceva?
Sì□ No□
2.2 Ci sono state difficoltà nell'inserimento? Sì□ No□ Se sì, quali? ______________________________
2.3 Partecipava volentieri alle attività in classe? Sì□ No□
2.4 Giocava volentieri con i coetanei? Sì□ No□
2.5 Durante il gioco, prendeva spesso l'iniziativa? Sì□ No□

3. Scuola elementare:

3.1 Frequenta una scuola a tempo pieno? Sì □ No□
3.2 Va volentieri a scuola? Sì □ No□
3.3 Attualmente gioca volentieri con i compagni? Sì□ No□
3.4 Ha avuto difficoltà tra la prima e la seconda elementare a imparare:
i giorni della settimana? Sì □ No□
i mesi dell'anno? Sì □ No□
a leggere le ore dall'orologio? Sì □ No□

3.5 Ha presentato difficoltà specifiche di lettura e/o ortografia fin dall’inizio dell'apprendimento? Sì ☐ No ☐
3.6 È autonomo nei compiti? Sì ☐ No☐ Se no, con chi li svolge?___________________________
3.7 Lo svolgimento dei compiti a casa richiede al bambino un tempo tale da impedirgli di dedicarsi ad interessi extrascolastici (es: sport, amicizie, gite con amici o famigliari, ecc..)? Sì ☐ No☐
3.8 Svolge abitualmente attività extrascolastiche e/o sport? Sì ☐ No☐
Se Si, quali? ____
3.9 Ha degli interessi particolari che coltiva attraverso la lettura? Sì ☐ No☐
4.0 Nell’ultimo mese ha letto almeno un libro? Sì ☐ No☐

4. Altri fattori di rilievo

4.1 Ha avuto traumi cranici di una certa importanza (ad esempio con perdita di conoscenza e/o necessità di una visita pediatrica o specialistica)? Sì ☐ No ☐
4.2 Ha avuto convulsioni febbrili? Sì☐ No ☐
4.3 Ha ricevuto diagnosi di epilessia? Sì☐ No ☐ Se sì, specificare ______________________________
4.4 Ha fatto visite oculistiche? Sì ☐ No ☐ Se sì, per quale motivo? _____________________________
4.5 Usa gli occhiali? Sì ☐ No☐ Se Si, per quale motivo? _____________________________________
4.6 Ha avuto otiti ricorrenti? Sì☐ No☐ 4.7 Ha avuto in passato problemi di udito? Sì ☐ No ☐
4.8 Ha presentato altre malattie di rilievo? Sì ☐ No ☐ Se si, quali?______________________________
4.9 Ci sono stati cambiamenti e/o eventi importanti nella vita di suo figlio? Sì ☐ No☐
Se Si, quali? Lutti ☐ In che anno di vita del bambino/a?_____ Nascite ☐ In che anno di vita del bambino/a? _____ Trasferimenti ☐ In che anno di vita del bambino/a? _____
Importanti cambiamenti lavorativi dei genitori ☐ In che anno di vita del bambino/a? _____
Altro_____________________In che anno di vita del bambino/a? _____
4.10 E’ già stato sottoposto a:

accertamenti per difficoltà di apprendimento?	Sì ☐	No ☐
riabilitazione per difficoltà di apprendimento?	Sì ☐	No ☐
visita o indagine psicologica per altre motivazioni?	Sì ☐	No ☐

4.11 Durante la prima infanzia (0-6 anni), c’è stato qualche adulto in famiglia che ha letto libri a suo figlio (libri adatti all’età del bambino, storie, racconti, fiabe ecc..)? Sì ☐ No ☐

La preghiamo ancora di rispondere ad alcune brevi domande sugli altri membri della famiglia

Padre del bambino: Età _______
Titolo di studio: Nessuno ☐ Elementare ☐
Media inferiore ☐ Media superiore ☐ Laurea ☐
Ha avuto un ritardo del linguaggio? Sì ☐ No ☐
Ha ripetuto una classe? Sì ☐ No ☐
Ha avuto difficoltà di apprendimento? Sì ☐ No ☐
Ha ricevuto una diagnosi di dislessia o disortografia? Sì ☐ No ☐
Ama leggere? Sì ☐ No☐
Ha presentato crisi epilettiche? Sì ☐ No☐

Madre del bambino: Età _______
Titolo di studio: Nessuno ☐ Elementare ☐
Media inferiore ☐ Media superiore ☐ Laurea ☐
Ha avuto un ritardo del linguaggio? Sì ☐ No ☐
Ha ripetuto una classe? Sì ☐ No ☐
Ha avuto difficoltà di apprendimento? Sì ☐ No ☐
Ha ricevuto una diagnosi di dislessia o disortografia? Sì ☐ No ☐
Ama leggere? Sì ☐ No☐
Ha presentato crisi epilettiche? Sì☐ No ☐

Eventuali fratelli:

Hanno avuto un ritardo del linguaggio?	Sì ☐	No ☐
Hanno avuto difficoltà di apprendimento?	Sì ☐	No ☐
Hanno ricevuto una diagnosi di dislessia o disortografia?	Sì ☐	No ☐
Hanno ripetuto una classe?	Sì ☐	No ☐
Hanno presentato crisi epilettiche?	Sì ☐	No ☐
